# Dietary habits and their association with irritable bowel syndrome among the public population in Saudi Arabia: a multicenter based study

**DOI:** 10.3389/fpubh.2026.1804238

**Published:** 2026-05-13

**Authors:** Shereen Ahmed A. Qalawa, Mohamed Goda Elbqry, Fatma Mohamed Elmansy, Aryam Tariq Almunif, Bashayr Falah Alharbi, Reema Jazzaa Alharbi, Joud Abdullah Alharbi, Kadi Matar Alharbi, Shaden Abdulaziz Almuhaysin, Shahad Turki Almutairi, Wajd Mohammed Albashir

**Affiliations:** 1 Department of Medical-Surgical Nursing, College of Nursing, Qassim University, Buraydah, Saudi Arabia; 2 Department of Nursing, Medical City, Qassim University, Buraydah, Saudi Arabia; 3 Bachelor of Science in Nursing, College of Nursing, Qassim University, Buraydah, Saudi Arabia

**Keywords:** cross-sectional study, dietary habits, eating behaviours, IBS symptom experience, irritable bowel syndrome, public health nutrition

## Abstract

**Background:**

Irritable bowel syndrome (IBS) is a prevalent functional gastrointestinal disorder affecting approximately 5.7% of the general population, with dietary factors frequently implicated in symptom exacerbation. Despite growing interest, evidence linking specific dietary behaviors to IBS symptom experience remains inconsistent.

**Aim:**

This study examines the associations between dietary habits and IBS symptom experience in adults and evaluates the relationship between sociodemographic characteristics and dietary behaviors.

**Methods:**

A descriptive correlational, multicenter study was conducted over a six-month period across several cities within the Qassim region, in accordance with the Strengthening the Reporting of Observational Studies in Epidemiology (STROBE) guidelines. A total of 552 adults aged ≥20 years were recruited using online convenience sampling. Data were collected using a structured, self-administered online questionnaire comprising three sections sociodemographic characteristics, IBS symptom experience using a symptom checklist, health history assessment, dietary habits and eating behaviours. Ethical approval was obtained prior to data collection, and informed consent was secured electronically. Participant confidentiality and data anonymity were strictly maintained throughout the study.

**Results:**

Among 552 participants, several dietary behaviors showed statistically significant but weak correlations with IBS symptom experience. Regular water intake (*ρ* = −0.16, *p* < 0.01), eating with family (*ρ* = −0.09, *p* = 0.03), and frequent consumption of grains and legumes (*ρ* = −0.08 to −0.09, *p* ≤ 0.05) were negatively associated with IBS symptoms, indicating a lower symptom burden. In contrast, eating in a hurry (*ρ* = 0.09, *p* = 0.05), frequent snacking (*ρ* = 0.09, *p* = 0.03), and higher intake of spicy foods (*ρ* = 0.08, *p* = 0.05) and sweets (*ρ* = 0.09, *p* = 0.01) were positively associated with IBS symptom severity. Sociodemographic factors also showed significant associations with dietary behaviors. Age demonstrated positive associations with healthier dietary patterns and negative associations with less healthy behaviors (e.g., hurried eating and sweets intake). Educational level showed positive associations with structured eating behaviors, whereas smoking status was positively associated with less healthy dietary behaviors.

**Conclusion:**

Unhealthy dietary habits were significantly associated with both the presence and severity of IBS symptoms among adults in the Qassim region. Sociodemographic and lifestyle factors further influenced these dietary behaviours. These findings emphasize the importance of patient-centred nutritional education, structured lifestyle modification programs, and individualized dietary interventions to enhance adherence and optimize IBS management.

## Introduction

Irritable bowel syndrome (IBS) is a common chronic functional gastrointestinal disorder characterized by recurrent abdominal pain associated with altered bowel habits, including constipation, diarrhoea, or a mixed pattern (1). Owing to its persistent symptoms, complex etiology, and negative impact on quality of life, IBS represents a significant clinical and public health concern. Globally, IBS affects approximately 5.7–10% of the population, with substantial geographic variation (2). Prevalence estimates range from 3.5–5.9% in Europe, 1.3–4.7% in Asia, 3.5–5.3% in North America, and up to 17.5% in Latin America, while rates in Saudi Arabia are notably higher, ranging between 14.4 and 22% (3).

IBS is classified under functional gastrointestinal disorders (FGIDs), which are defined by chronic gastrointestinal symptoms in the absence of identifiable structural abnormalities (4). Its pathophysiology is multifactorial, involving disturbances in gut motility, visceral hypersensitivity, the gut–brain axis, immune activation, gut microbiota composition, and food-related sensitivities. These mechanisms contribute to symptom chronicity, psychological comorbidities, increased healthcare utilization, and impaired quality of life (5).

Dietary habits are among the most important and modifiable factors influencing IBS symptoms. Evidence indicates that 85–90% of individuals with IBS associate symptom exacerbation with specific foods. High-fat foods, fermentable carbohydrates, caffeine, alcohol, and spicy foods are frequently reported triggers, whereas dietary fibre particularly soluble may alleviate symptoms in some patients (6). Dietary effects on IBS are mediated through fermentation, gas production, osmotic activity, immune modulation, and alterations in gut microbiota (4). In Saudi Arabia, rapid nutritional transitions toward Western-style dietary patterns characterized by high intake of saturated fats, refined carbohydrates, and processed foods have coincided with rising obesity rates and diet-related chronic conditions. Despite national and World Health Organization–supported initiatives promoting healthier dietary behaviours, adherence remains suboptimal (7).

Nurses play a central role in IBS management through patient education, dietary counselling, and support for lifestyle modification. Nursing-led interventions emphasize regular meal patterns, adequate hydration, appropriate fibre intake, stress management, and physical activity, all of which are essential for symptom control and long-term self-management (6). Understanding patient dietary preferences and real-world adherence behaviours is therefore critical for designing effective, patient-centred nursing interventions. Accordingly, this study aimed to examine the association between dietary habits and irritable bowel syndrome among adults in the Qassim region of Saudi Arabia (8).

The conceptual framework of this study is based on the premise that dietary habits play a dual role in IBS, acting both as symptom triggers and as therapeutic tools. Given the complex and multifactorial nature of IBS, management strategies often focus on symptom control rather than cure. Many patients identify specific foods such as coffee, alcohol, spicy foods, and high-fat meals as symptom triggers, and dietary avoidance of these items may reduce symptom severity (9). Within this framework, patient dietary preferences, lifestyle behaviours, and adherence to dietary recommendations directly influence IBS symptom severity and quality of life (1). Nursing interventions serve as a mediating factor by providing individualized dietary guidance, promoting healthy lifestyle behaviours, and supporting sustained adherence. This patient-centred approach aligns with contemporary IBS management models and emphasizes the importance of preference-sensitive, adherence-focused care (7).

### Research aim

This study examines the association between dietary habits and IBS symptom experience in adults and evaluates the relationship between sociodemographic characteristics and dietary behaviors.

## Methods

### The study design and setting

A descriptive correlational, multicenter study was conducted over a period of approximately 4–6 months across multiple cities in the Qassim region of Saudi Arabia.

### Sampling and population

A convenience sampling technique was used to recruit adults residing in the Qassim region who were available during the data collection period. Adults aged 20 years and above, of both sexes, with or without a prior diagnosis of irritable bowel syndrome (IBS), were eligible to participate, while individuals younger than 20 years, those with cognitive impairment, or those who participants who were unable to provide informed consent due to cognitive or severe psychiatric impairment were excluded. Sample size estimation was conducted using Raosoft® sample size calculation software based on the estimated adult population of the Qassim region derived from official sociodemographic statistics, assuming a 95% confidence level, 80% statistical power, a 5% margin of error, and an anticipated 5% non-response or dropout rate. The required sample size was calculated using the standard cross-sectional formula $n=(Z^{2}\times p\times (1-p))/d^{2}$ , where the Z-value for a 95% confidence level was 1.96, the estimated population proportion was set at 0.50, and the margin of error was 0.05. After adjustment for the finite population and expected dropout, the final sample size was recalculated using Epi Info™ version 7.2.5.0, resulting in a total of 552 participants included in the study.

### Data collection tools

Data were collected using an online structured, self-administered questionnaire created via Google Forms and distributed within the Qassim region. The questionnaire was adapted from previously validated instruments used in related studies (6–11, 12) and comprised three main sections.

1. The first section collected sociodemographic, including age, gender, nationality, educational level, and smoking status.
2. The second section functioned as a structured health history assessment. A structured IBS-related symptom checklist, derived from selected items of the IBS Symptom Severity Scoring System (IBS-SSS), was used to assess IBS symptom experience as the outcome variable, rather than to establish a formal clinical diagnosis. No diagnostic criteria (e.g., Rome IV) or IBS-SSS cut-off scores were applied. The assessment included key gastrointestinal symptoms, namely flatus, anorexia, urgency, bloating, diarrhea, tenesmus, presence of mucus in stool, abdominal pain, and nocturnal bowel movements. A composite IBS symptom experience score was calculated by summing responses to these nine symptom items, each measured on a 3-point Likert scale (0–2), yielding a total score ranging from 0 to 18, with higher scores indicating greater symptom burden. Participants also self-reported existing medical conditions, including prior IBS symptom experience, family history of gastrointestinal disorders, hypertension, diabetes mellitus, thyroid disease, anemia, and other relevant conditions.
3. The third section assessed dietary habits and eating behaviors. This section evaluated personal dietary practices using food frequency items focused on commonly consumed foods relevant to gastrointestinal health, including whole grains (such as oats and brown rice), leafy vegetables, lean protein sources, fresh fruits, nuts and seeds, and low-fat dairy products. Additional dietary behaviors including breakfast consumption, number of meals and snacks per day, frequency of home-cooked meals and family meals, and eating speed were assessed using a three-point Likert ranging from 0 (rarely), 1 (sometimes) to 2 (always).

### The study protocol

Participants were recruited through an online study protocol conducted within the Qassim region of Saudi Arabia, which served as the study field. The protocol was designed to ensure transparency, informed consent, and voluntary participation. At the start of the survey, a brief introduction outlining the study aim, objectives, and significance was provided. It was clearly stated that participation was voluntary, responses were anonymous, and all data would be used solely for research purposes. The estimated completion time of the questionnaire (approximately 3–5 min) was indicated, and informed consent was implied by participants’ decision to proceed with the survey. Data were collected using a structured questionnaire administered via Google Forms, and the survey link was distributed through community websites and public online forums accessible to residents of the Qassim region. Data collection was conducted from May to October 2025.

### Pilot study

A pilot study was conducted prior to the main data collection to assess the clarity, feasibility, and completion time of the questionnaire. The pilot included approximately 10% of the estimated sample size, selected from the target population and meeting the same inclusion criteria. The average completion time was approximately 3–5 min, indicating good feasibility for online administration. Participants in the pilot study were not included in the final analysis. Minor modifications were made based on participant feedback to improve item wording and questionnaire flow.

### Validity and reliability

Content validity of the questionnaire was ensured through adaptation from previously validated instruments reported in the literature. Face and content validity were further reviewed by a panel of experts in medical-surgical nursing, community health nursing, and gastroenterology, who evaluated the relevance, clarity, and appropriateness of the items. Necessary revisions were incorporated based on expert feedback. Internal consistency reliability assessed using Cronbach’s alpha coefficient. The overall questionnaire demonstrated acceptable reliability, with Cronbach’s alpha values exceeding 0.75 and 0.70 for all major domains, including A structured IBS-related symptom checklist and dietary habits and IBS symptoms, respectively.

### Statistical analysis

Data were coded, cleaned, and analyzed using the Statistical Package for the Social Sciences (SPSS) version 26.0. Descriptive statistics were used to summarize participant characteristics and study variables, including frequencies, percentages, means, and standard deviations, as appropriate. Inferential statistical analyses were conducted to examine associations between dietary habits, sociodemographic variables, and IBS outcomes. The chi-square test was used to assess associations between categorical variables, while independent sample *t*-tests and one-way analysis of variance (ANOVA) were applied to compare mean IBS-SSS scores across different groups. Pearson’s correlation coefficient was used to examine relationships between continuous variables. All statistical tests were two-tailed, and a *p*-value of <0.05 was considered statistically significant.

## Results

Table 1 presents that the sample was predominantly young, with the largest proportion aged 20–25 years (38.9%), although all adult age groups were represented. Females comprised slightly more than half of the participants (55.3%). The majority of respondents were Saudi nationals (90.7%), reflecting the sociodemographic composition of the study region. In terms of educational attainment, most participants were university educated (73.9%), indicating a relatively high educational level within the sample. Smoking prevalence was low, with only 3.4% reporting current smoking. Regarding place of residence, 38.6% of participants resided within Buraydah, while 61.4% lived in areas outside Buraydah, suggesting broad geographic representation across the Qassim region.

**Table 1:** Distribution of sociodemographic characteristics among study participants (*n* = 552).

Variable	Category	No (%)
Age	20 < 25	215 (38.9%)
	25 < 30	38 (6.9%)
	30 < 35	38 (6.9%)
	35 < 40	53 (9.6%)
	40 < 45	66 (12.0%)
	45 < 50	75 (13.6%)
	≥50	67 (12.1%)
Gender	Male	247 (44.7%)
	Female	305 (55.3%)
Nationality	Saudi	501(90.7%)
	Non-Saudi	52 (9.3%)
Education level	University	408 (73.9%)
	Tertiary	110 (19.9%)
	Read and write	9 (1.6%)
	Primary	5 (0.9%)
	Secondary	20 (3.6%)
Smoking status	Yes	19 (3.4%)
	No	533 (96.6%)
Residence place	Inside Buraydah	213 (38.6%)
	Outside Buraydah	339 (61.4%)

**Figure 1:**
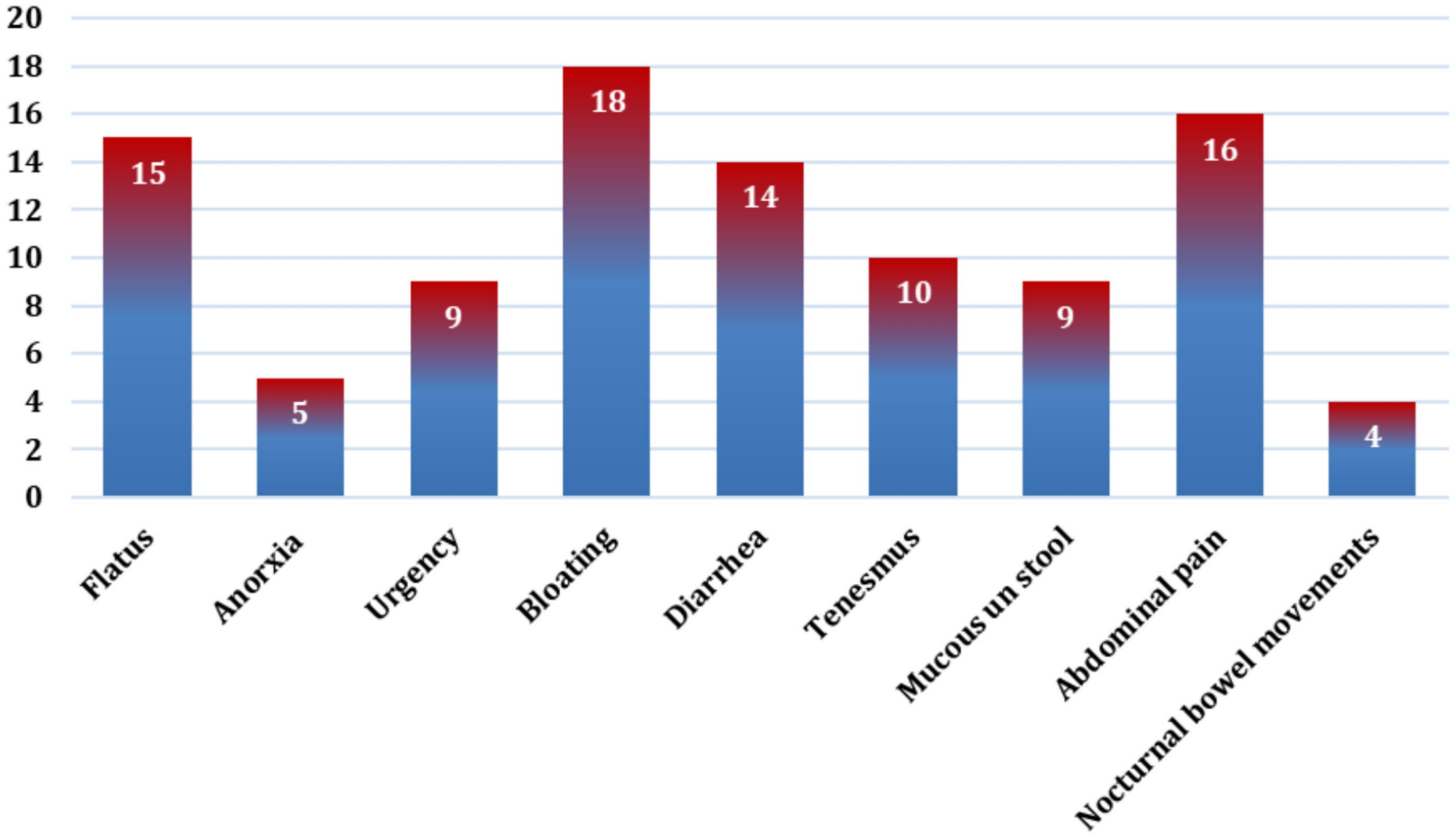
Distribution of the study participants according to their IBS symptom experience (*n* = 552).

Figure 1 illustrates that the bloating was the most frequently reported symptom, affecting 18% of participants, followed by abdominal pain (16%) and flatus (15%). Diarrhea was reported by 14%, while tenesmus was experienced by 10% of participants. Both urgency and mucus in stool were reported by 9% of the sample. Less frequently reported symptoms included anorexia (5%) and nocturnal bowel movements (4%). Overall, the figure demonstrates that abdominal pain–related symptoms, particularly bloating and abdominal pain, represent the predominant clinical complaints among participants experiencing IBS symptoms.

**Figure 2:**
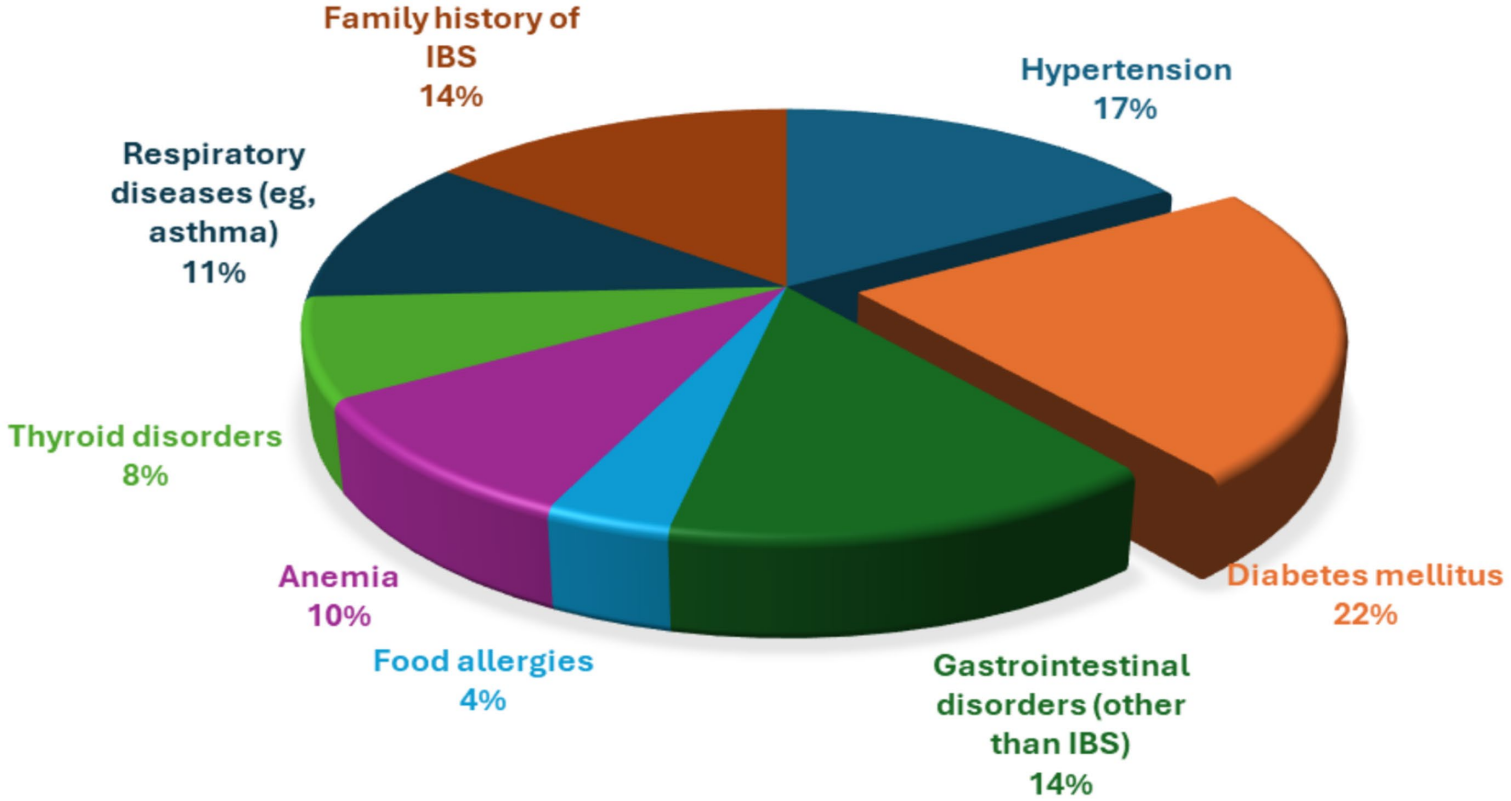
Distribution of self-reported health conditions among study participants (*n* = 552).

Figure 2 reveals that the diabetes mellitus was the most frequently reported condition, affecting 22% of participants, followed by hypertension (17%). Gastrointestinal disorders other than irritable bowel syndrome (IBS) and a family history of IBS were each reported by 14% of participants. Respiratory diseases, including asthma, accounted for 11%, while anemia and thyroid disorders were reported by 10 and 8%, respectively. Food allergies were the least frequently reported condition, affecting 4% of the study population.

Table 2 presents that slightly more than half of the participants reported drinking water regularly (51.6%) and eating meals regularly (55.9%). Breakfast consumption was relatively common, with 59.8% reporting always eating breakfast, while 11.2% rarely consumed breakfast. Regarding snack intake, the majority of participants reported consuming two to three snacks per day sometimes (52.5%), whereas only 16.3% reported doing so regularly. Home-cooked meals were frequently consumed, with 54.3% of participants reporting regular intake, and eating with family was common, as 83.4% reported doing so always or sometimes. However, eating behaviours that may negatively affect gastrointestinal health were prevalent; 35.8% reported always eating in a hurry and 54.7% reported doing so sometimes.

**Table 2:** Distribution of dietary habits among the study participants (*n* = 552).

Dietary habit	Response category	No (%)
1. How often do you drink water regularly?	Always	285 (51.6%)
	Sometimes	96 (17.5%)
	Rarely	171 (30.9%)
2. How often do you eat meals regularly?	Always	309 (55.9%)
	Sometimes	62 (11.3%)
	Rarely	181 (32.8%)
3. How often do you eat breakfast?	Always	330 (59.8%)
	Sometimes	160 (29.0%)
	Rarely	62 (11.2%)
4. How often do you consume two to three snacks per day?	Always	90 (16.3%)
	Sometimes	290 (52.5%)
	Rarely	172 (31.2%)
5. How often do you consume home-cooked meals?	Always	300 (54.3%)
	Sometimes	200 (36.2%)
	Rarely	52 (9.5%)
6. How often do you eat with your family?	Always	230 (41.7%)
	Sometimes	230 (41.7%)
	Rarely	92 (16.7%)
7. How often do you eat in a hurry?	Always	198 (35.8%)
	Sometimes	302 (54.7%)
	Rarely	52 (9.5%)
8. How often do you consume caffeinated beverages?	Always	356 **(64.5%)**
	Sometimes	191 **(34.6%)**
	Rarely	5 **(0.9%)**
9. How often do you consume canned foods?	Always	350 **(63.4%)**
	Sometimes	150 **(27.2%)**
	Rarely	52 **(9.4%)**
10. How often do you consume spicy foods?	Always	300 **(54.3%)**
	Sometimes	150 **(27.2%)**
	Rarely	102 **(18.5%)**
11. How often do you consume milk per week?	Always	200 **(36.2%)**
	Sometimes	250 **(45.3%)**
	Rarely	102 **(18.5%)**
12. How often do you consume grains, pasta, or cereals per week?	Always	300 (54.3%)
	Sometimes	180 (32.6%)
	Rarely	72 (13.1%)
13. How often do you consume sweets and desserts?	Always	100 (18.1%)
	Sometimes	250 (45.3%)
	Rarely	202 (36.6%)
14. How often do you eat late at night (after 10:00 p.m.)?	Always	200 (36.2%)
	Sometimes	290 (52.5%)
	Rarely	62 (11.3%)
15. How often do you consume legumes (beans, lentils, etc.) per week?	Always	250 **(45.3%)**
	Sometimes	220 **(39.9%)**
	Rarely	82 (14.9%)

Caffeinated beverage consumption was high, with 64.5% reporting regular intake and only 0.9% rarely consuming caffeine. Processed food consumption was also notable, as 63.4% of participants reported regularly consuming canned foods and 54.3% reported frequent consumption of spicy foods. Milk consumption varied, with most participants reporting occasional (45.3%) or regular (36.2%) intake. More than half of the participants reported regular consumption of grains, pasta, or cereals (54.3%), while legumes were consumed regularly by 45.3% of participants. Consumption of sweets and desserts was mostly occasional (45.3%), although 18.1% reported daily intake. Late-night eating was relatively common, with 36.2% reporting frequent consumption of meals after 10:00 p.m. and 52.5% reporting doing so sometimes.

Table 3 shows that regular water consumption was significantly negatively correlated with IBS symptom experience (*ρ* = −0.16, *p* < 0.01). Similarly, eating with family (*ρ* = −0.09, *p* = 0.03), consumption of grains, pasta, or cereals (*ρ* = −0.08, *p* = 0.05), canned food consumption (*ρ* = −0.08, *p* = 0.04), and legume intake (*ρ* = −0.09, *p* = 0.03) were also negatively associated with IBS symptoms. These correlations were weak in magnitude and should be interpreted with caution. While some findings (e.g., grains and legumes) may suggest a potentially beneficial role of structured meals and fiber-rich dietary components, others (e.g., canned food consumption) may reflect residual confounding or population-specific dietary patterns rather than true protective effects.

**Table 3:** Relationship between dietary habits and the experience of IBS symptoms (*n* = 552).

Dietary habit item	IBS symptom experience	
	Spearman’s correlation (ρ)	Sig. (2-tailed)
1. How often do you eat breakfast?	0.07*	0.03
2. How often do you eat in a hurry?	0.09*	0.05
3. How often do you eat meals regularly?	0.02*	0.03
4. How often do you consume spicy foods?	0.08*	0.05
5. How often do you eat with your family?	−0.09*	0.03
6. How often do you drink water regularly?	−0.16**	<0.01
7. How often do you consume canned foods?	−0.08*	0.04
8. How often do you consume milk per week?	−0.02	0.26
9. How often do you consume sweets and desserts?	0.09*	0.01
10. How often do you consume home-cooked meals?	−0.03	0.35
11. How often do you consume caffeinated beverages?	−0.02	0.40
12. How often do you eat late at night (after 10:00 p.m.)?	−0.03	0.40
13. How often do you consume two to three snacks per day?	0.09*	0.03
14. How often do you consume grains, pasta, or cereals per week?	−0.08*	0.05
15. How often do you consume legumes (beans, lentils, etc.) weekly?	−0.09*	0.03

*Correlation is significant at the 0.05 level (2-tailed).**Correlation is significant at the 0.01 level (2-tailed).

In contrast, several eating behaviors showed weak positive correlations with IBS symptom experience. Regular meal consumption (*ρ* = 0.02, *p* = 0.03), breakfast intake (*ρ* = 0.07, *p* = 0.03), consumption of two to three snacks per day (*ρ* = 0.09, *p* = 0.03), eating in a hurry (*ρ* = 0.09, *p* = 0.05), spicy food consumption (*ρ* = 0.08, *p* = 0.05), and intake of sweets and desserts (*ρ* = 0.09, *p* = 0.01) were significantly associated with higher IBS symptom scores. No statistically significant associations were observed between IBS symptom experience and consumption of home-cooked meals (*p* = 0.35), caffeinated beverages (*p* = 0.40), milk intake (*p* = 0.26), or late-night eating (*p* = 0.40).

Table 4 shows that age was significantly associated with several dietary behaviours. Older age was positively correlated with regular water consumption (*r* = 0.14, *p* = 0.01) and negatively correlated with regular meal consumption (*r* = −0.18, *p* < 0.01), eating in a hurry (*r* = −0.20, *p* < 0.01), consumption of canned foods (*r* = −0.22, *p* = 0.05), milk intake (*r* = −0.11, *p* = 0.04), and intake of sweets and desserts (*r* = −0.12, *p* = 0.05). Overall, these findings indicate that older participants tended to report more structured and potentially healthier dietary behaviours, although the correlations were weak in magnitude. Gender was also significantly associated with certain dietary behaviours. Female participants were less likely to report regular water intake (*r* = −0.09, *p* = 0.02), regular meal consumption (*r* = −0.08, *p* = 0.04), and eating in a hurry (*r* = −0.15, *p* < 0.01), while no significant associations were observed with most food group consumption patterns.

**Table 4:** Relationship between sociodemographic characteristics among study participants and their dietary habits (*n* = 552).

Item	Age		Gender		Education		Smoking	
	*r*	*p*	*r*	*p*	*r*	*p*	*r*	*p*
1. How often do you drink water regularly?	0.14**	0.01	−0.09*	0.02	0.01	0.80	−0.09*	0.02
2. How often do you eat meals regularly?	−0.18**	<0.01	−0.08*	0.04	0.05	0.20	−0.01	0.76
3. How often do you eat breakfast?	−0.06	0.14	0.02	0.62	0.1**	0.01	0.10*	0.01
4. How often do you consume two to three snacks per day?	−0.09*	0.02	−0.04	0.92	0.09*	0.02	−0.01	0.68
5. How often do you consume home-cooked meals?	−0.11	0.06	0.05	0.20	0.02	0.54	0.05	0.18
6. How often do you eat with your family?	0.03	0.38	−0.03	0.38	0.02	0.49	−0.08	0.06
7. How often do you eat in a hurry?	−0.20**	<0.01	−0.15**	<0.01	0.03	0.48	−0.08	0.06
8. How often do you consume caffeinated beverages?	−0.06	0.12	0.05	0.31	0.06	0.07	0.07*	0.03
9. How often do you consume canned foods?	−0.22**	0.05	0.04	0.21	0.02	0.51	0.04	0.19
10. How often do you consume spicy foods?	0.02	0.42	−0.02	0.37	0.03	0.40	−0.07	0.05
11. How often do you consume milk per week?	−0.11**	0.04	0.05	0.21	0.03	0.42	0.04	0.14
12. How often do you consume grains, pasta, or cereals per week?	0.02	0.41	−0.02	0.33	0.09	0.21	−0.09	0.07
13. How often do you consume sweets and desserts?	−0.12**	0.05	0.04	0.28	0.08	0.45	0.06	0.19
14. How often do you eat late at night (after 10:00 p.m.)?	0.04	0.36	−0.02	0.39	0.04	0.40	−0.07	0.07
15. How often do you consume legumes (beans, lentils, etc.) per week?	−0.12	0.06	0.04	0.21	0.06	0.50	0.04	0.13

*r*, Pearson’s correlation coefficients. *Correlation is significant at the 0.05 level (2-tailed).

**Correlation is significant at the 0.01 level (2-tailed).

Educational level showed weak positive correlations with breakfast consumption (*r* = 0.10, *p* = 0.01) and snack intake (*r* = 0.09, *p* = 0.02), suggesting that individuals with higher education levels may have more structured eating patterns. However, no significant associations were observed with most other dietary components. Smoking status was positively associated with breakfast consumption (*r* = 0.10, *p* = 0.01) and caffeinated beverage intake (*r* = 0.07, *p* = 0.03). These findings may reflect behavioural patterns among smokers that could influence gastrointestinal health, although the observed associations were weak. No significant relationships were found between smoking status and most other dietary behaviours.

## Discussion

This multicenter study examined the associations between dietary habits and IBS symptom experience among adults in the Qassim region. The findings indicate that several dietary behaviors were significantly associated with IBS symptom experience; however, these associations were generally weak in magnitude and should be interpreted with caution. While IBS is widely acknowledged as a multifactorial disorder influenced by psychological stress, sleep disturbances, and genetic predisposition, dietary behavior remains an important and modifiable factor linked to gastrointestinal physiology (1–13). However, the present findings should be interpreted as associational rather than causal, and do not imply that dietary habits directly determine IBS symptom experience. Importantly, due to the cross-sectional design, causal inferences cannot be established. The observed relationships may reflect bidirectional effects, where dietary behaviors influence symptoms, while individuals experiencing symptoms may also modify their dietary patterns (reverse causality).

### Sociodemographic profile

The sociodemographic profile of the current study reflects a predominantly young, educated, and female population, which is consistent with several regional and national studies conducted among adult and community-based samples in Saudi Arabia and the Gulf region. The high proportion of participants aged 20–25 years may be attributed to the widespread use of online data-collection methods, which tend to attract younger and more technologically engaged individuals, as reported previously (14). Similarly, the predominance of female participants aligns with earlier studies indicating higher response rates among women in health-related surveys, possibly due to greater health awareness and engagement (15). The high percentage of Saudi nationals and university-educated respondents mirrors the demographic characteristics of the Qassim region (16); however, it may also reflect the influence of convenience sampling, which can affect representativeness.

The relatively low smoking prevalence observed in this study is lower than that reported in some national surveys (17), which may be explained by the younger age distribution and higher educational level of the participants. Conversely, the broad geographic distribution, with more than half of participants residing outside Buraydah, enhances regional coverage and is consistent with multicenter regional studies (18). However, the relatively high educational attainment and reliance on an online convenience sample may introduce selection bias, particularly by underrepresenting individuals with limited digital access or lower educational levels. Therefore, caution is warranted when generalizing these findings to more socioeconomically diverse populations.

### IBS manifestations

The predominance of bloating and abdominal pain observed in the present study is consistent with the established clinical profile of irritable bowel syndrome (IBS), in which abdominal pain–related symptoms are commonly reported as key features. Similar findings have been reported in regional and international studies identifying bloating, abdominal discomfort, and altered bowel habits as the most frequently experienced symptoms, particularly among young adults (14).

The relatively high frequency of diarrhea and flatus in this sample is also consistent with reports suggesting that mixed and diarrhea-predominant symptom patterns are common in community-based populations (19). Conversely, the lower frequency of nocturnal bowel movements and anorexia aligns with previous evidence indicating that these symptoms are less typical of functional bowel disorders and may warrant further evaluation when present (9).

It is important to note that the current study assessed IBS symptom experience rather than clinical diagnosis, and therefore the findings reflect patterns of reported symptoms rather than confirmed IBS subtypes. Overall, the observed symptom distribution highlights the heterogeneity of IBS-related symptoms and underscores the importance of symptom-focused assessment and individualized management strategies in clinical and nursing practice.

### Self-reported health conditions

The distribution of self-reported health conditions in the present study indicates a substantial burden of chronic diseases, particularly diabetes mellitus and hypertension, which is consistent with national epidemiological data reporting high prevalence rates of metabolic and cardiovascular conditions among adults in Saudi Arabia.

The coexistence of other gastrointestinal conditions and a family history of IBS-related symptoms support existing evidence suggesting that gastrointestinal symptom patterns often overlap and may be influenced by familial or genetic factors (20). The prevalence of respiratory diseases, anaemia, and thyroid disorders observed in this study is comparable to findings from previous community-based research, suggesting that these comorbidities may influence gastrointestinal symptom experience through inflammatory, hormonal, or psychosomatic pathways (13).

In contrast, the relatively low prevalence of food allergies differs from some international studies reporting stronger associations between perceived food intolerance and gastrointestinal symptoms, which may be attributable to variations in diagnostic criteria, cultural dietary practices, or reliance on self-reported data (7). Given the cross-sectional design and reliance on self-reported conditions, these associations should be interpreted with caution, as causal relationships cannot be established and reporting bias may be present. Overall, these findings underscore the importance of incorporating a comprehensive assessment of comorbid chronic conditions when evaluating individuals with IBS symptom experience, as multimorbidity may contribute to symptom complexity and influence clinical management and patient-centred care.

### Dietary habits and eating behaviors of the study participants

The dietary habits observed in this study reflect a mixed pattern of both potentially beneficial and less favorable eating behaviors. Although more than half of the participants reported regular water intake, meal regularity, and frequent breakfast consumption, these findings are only partially consistent with previous studies suggesting that irregular meal patterns and breakfast skipping are common among young adults and may be associated with functional gastrointestinal symptoms, including IBS (7–9, 11, 12).

The high frequency of home-cooked meals and eating with family reflects culturally rooted dietary practices in Saudi Arabia and has been previously associated with improved dietary quality and better gastrointestinal health (7–9, 11, 12). However, several eating behaviors identified in this study, including eating in a hurry, frequent consumption of caffeinated beverages, spicy foods, and processed or canned foods, have been reported in prior studies as factors associated with increased gastrointestinal symptom burden in IBS populations (20).

The high prevalence of caffeine intake observed in this study exceeds that reported in some international cohorts (21), which may reflect regional preferences for coffee and tea consumption. Similarly, late-night eating was commonly reported and is consistent with prior regional findings linking such behaviors to altered gastrointestinal function and increased symptom reporting (19). Conversely, the relatively frequent intake of grains, legumes, and milk suggests a degree of dietary diversity; however, tolerance to these foods may vary considerably among individuals experiencing IBS-related symptoms (22). Differences between the current findings and some Western studies may be attributed to variations in cultural dietary patterns, food availability, and the reliance on self-reported dietary behaviors (23).

Importantly, the associations observed in this study were generally weak in magnitude, indicating limited clinical relevance despite statistical significance. Furthermore, given the cross-sectional design and reliance on self-reported dietary behaviors, causal relationships cannot be established. The observed patterns may reflect bidirectional relationships, where dietary habits influence symptom experience, while individuals with symptoms may also modify their eating behaviors (reverse causality). Overall, these findings highlight the complex and multifactorial nature of dietary influences on gastrointestinal health and underscore the importance of individualized dietary assessment and patient-centered nutritional counseling in individuals experiencing IBS-related symptoms.

### Association between dietary habits and IBS symptom experience

The findings of the present study demonstrate statistically significant associations between specific dietary habits and IBS symptom experience; however, these associations were generally weak in magnitude, suggesting limited clinical relevance despite statistical significance. These results highlight the complex and potentially bidirectional relationship between eating behaviours and gastrointestinal symptom experience. Regular water consumption was inversely associated with IBS symptom experience, which is consistent with previous studies suggesting that adequate hydration may support bowel function and is associated with reduced gastrointestinal discomfort in functional gastrointestinal disorders (15). Similarly, eating with family and the consumption of grains, cereals, and legumes were negatively correlated with IBS symptoms, which may reflect the role of structured eating patterns and fibre-containing foods in gastrointestinal health when tolerated (24).

In contrast, several eating behaviours, including frequent snacking, eating in a hurry, spicy food consumption, and intake of sweets and desserts, were positively associated with IBS symptom experience. These findings are generally consistent with prior literature reporting associations between certain dietary patterns and increased gastrointestinal symptom reporting (21). However, the small effect sizes observed in this study indicate that these factors alone are unlikely to have substantial clinical impact. Interestingly, some findings were counterintuitive. For example, regular meal consumption and breakfast intake were positively associated with IBS symptom experience. These patterns may reflect reverse causality, whereby individuals experiencing gastrointestinal symptoms adopt more structured eating habits in response to symptom onset rather than as a causal factor (23). Similar observations have been reported in previous cross-sectional studies, highlighting the limitations of such designs in establishing causal directionality (7).

Furthermore, the weak negative association observed with canned food consumption appears inconsistent with existing literature linking processed foods to gastrointestinal symptoms. Given the small magnitude of this association, it should be interpreted cautiously and may reflect residual confounding, reporting bias, or population-specific dietary patterns, rather than a true protective effect.

Notably, no significant associations were found between IBS symptom experience and consumption of home-cooked meals, caffeinated beverages, milk intake, or late-night eating. These findings partially contrast with some international studies reporting caffeine and dairy as common symptom triggers (25) but are consistent with others suggesting substantial interindividual variability in food tolerance among individuals experiencing IBS-related symptoms (12). Cultural dietary practices, adaptation to habitual intake, and reliance on self-reported dietary behaviours may further contribute to these differences.

Importantly, given the cross-sectional design and reliance on self-reported dietary behaviours, causal relationships cannot be established. The observed associations should therefore be interpreted as correlational, and future longitudinal or interventional studies are needed to better clarify the direction and clinical significance of these relationships. Overall, the findings underscore the heterogeneity of dietary influences on IBS symptom experience and reinforce the importance of individualized dietary assessment and patient-centred nutritional counselling rather than universal dietary restrictions.

### Relationship between sociodemographic characteristics and dietary habits

The findings of the present study demonstrate statistically significant associations between sociodemographic characteristics particularly age, gender, educational level, and smoking status—and dietary habits; however, these associations were generally weak in magnitude, suggesting limited clinical relevance despite statistical significance. Increasing age was associated with more structured eating behaviors, including higher water consumption and lower frequencies of hurried eating, processed food intake, sweets, and dairy products. These patterns are consistent with previous research indicating that older adults tend to adopt more cautious dietary practices, possibly due to greater health awareness or prior exposure to chronic conditions (24). Similarly, the inverse association between age and hurried eating aligns with evidence that younger individuals are more likely to engage in time-constrained or irregular eating patterns (25).

Gender-related differences were also observed. Female participants reported lower frequencies of regular water intake, meal regularity, and hurried eating. While the lower prevalence of hurried eating among females is consistent with reports of greater dietary mindfulness (26), the lower water intake and meal regularity contrast with some studies suggesting healthier patterns among females (27). These mixed findings may reflect cultural, occupational, or lifestyle factors specific to the study population. Educational level showed modest positive associations with breakfast consumption and snack intake, suggesting a potential link between education and awareness of meal timing and dietary routines. This is in line with prior studies identifying education as an important determinant of dietary behavior and nutritional knowledge (28). However, the lack of consistent associations with most food groups indicates that education alone may not fully explain overall dietary patterns.

Smoking status was positively associated with breakfast consumption and caffeine intake, which is consistent with literature describing clustering of lifestyle behaviors, including smoking and stimulant use (29, 30). The absence of significant associations between smoking and most dietary components may be influenced by the relatively low prevalence of smokers in the present example. Importantly, given the cross-sectional design and reliance on self-reported behaviors, these associations should be interpreted as correlational rather than causal. The observed patterns may reflect bidirectional relationships, where sociodemographic factors influence dietary behaviors, while lifestyle adaptations may also occur in response to health conditions or symptom experience. Overall, these findings highlight the multifactorial and context-dependent nature of dietary behaviors and underscore the importance of tailored, population-specific nutritional and lifestyle interventions.

### The study limitations

The descriptive correlational design limits causal inference, as dietary behaviours may both influence and be influenced by IBS symptom experience. The reliance on self-reported data introduces the potential for recall and reporting bias. Furthermore, the use of convenience sampling may restrict the generalizability of findings beyond the Qassim region. Nevertheless, the relatively large sample size and multicenter recruitment strengthen the robustness, relevance, and practical applicability of the results, particularly for informing nursing practice and community-based IBS management strategies.

Use of convenience sampling through an online questionnaire may have introduced selection bias, as individuals with limited digital literacy or restricted internet access were less likely to participate. This may have influenced the sociodemographic distribution of the sample and limits the generalizability of the findings to the broader population.

### Clinical practice implications

The findings of this study underscore the importance of integrating individualized dietary and lifestyle assessment into the routine clinical management of patients experiencing IBS symptoms. The observed associations between dietary habits and symptom experience highlight the need for tailored nutritional counselling rather than generalized dietary restrictions. Clinicians, particularly nurses, should support patients in identifying personal symptom triggers, promoting adequate hydration, encouraging mindful and unhurried eating, and reinforcing balanced intake of tolerated fibre-containing foods. The influence of sociodemographic characteristics on dietary behaviours suggests that age, gender, educational level, and smoking status should be considered when designing patient education and self-management plans. Younger individuals and those exhibiting maladaptive eating behaviours, such as hurried eating and frequent consumption of spicy foods and sweets, may benefit from targeted behavioural interventions. The variability in associations between IBS symptoms and commonly restricted foods further supports a patient-centred approach that emphasizes individual tolerance, shared decision-making, and sustained adherence.

## Conclusion and recommendations

This study identified statistically significant associations between dietary habits and IBS symptom experience among adults in the Qassim region; however, these associations were generally weak in magnitude and should be interpreted with caution. Unbalanced eating behaviours, including lower intake of fibre-rich foods, frequent consumption of spicy or processed foods, irregular meals, and hurried eating, were associated with greater symptom experience. Sociodemographic factors such as age, education level, smoking status, and nationality were also associated with dietary patterns, reflecting the multifactorial and context-dependent nature of IBS-related symptoms. Importantly, given the cross-sectional design and reliance on self-reported data, causal relationships cannot be established, and the observed associations may reflect bidirectional influences.

These findings support the potential value of integrating individualized and culturally sensitive dietary and lifestyle counselling into routine nursing care. Nurse-led interventions focusing on hydration, mindful eating, balanced fibre intake, and identification of personal dietary triggers may help improve symptom management and adherence. Future longitudinal and interventional studies are needed to better clarify the directionality of these associations and to evaluate the effectiveness of culturally tailored nurse-led dietary and behavioural programs on IBS symptom experience and quality of life.

## Data Availability

The original contributions presented in the study are included in the article/supplementary material, further inquiries can be directed to the corresponding author.
